# Serum tartrate-resistant acid phosphatase 5b activity as a prognostic marker of survival in breast cancer with bone metastasis

**DOI:** 10.1186/1471-2407-10-158

**Published:** 2010-04-23

**Authors:** Yi-Ying Wu, Anthony J Janckila, Chih-Hung Ku, Cheng-Ping Yu, Jyh-Cherng Yu, Su-Hui Lee, Hsin-Yi Liu, Lung T Yam, Tsu-Yi Chao

**Affiliations:** 1 Breast Cancer Research Group, Tri-Service General Hospital, National Defense Medical Center, Taipei, Taiwan; 2 Division of Hematology, Veterans Administrative Medical center, Louisville, Kentucky, USA; 3 Department of Public Health, National Defense Medical Center, Taipei, Taiwan; 4 National Institute of Cancer Research, National Health Research Institutes, Taipei, Taiwan

## Abstract

**Background:**

Serum tartrate-resistant acid phosphatase 5b (TRACP 5b) activity is a marker of osteoclast number and is elevated in breast cancer (BC) patients with extensive bone metastasis, which might in turn reflect the tumour burden. We tested the hypothesis that baseline serum TRACP 5b activity and its interval change are potential prognostic markers of survival in BC patients with bone metastasis.

**Methods:**

We analyzed the data from previous prospective studies. A total of 100 patients with newly diagnosed bone metastasis were included. Cox proportional regression model was used to evaluate the correlation between the overall survival time (OS) and baseline serum TRACP 5b activity and its interval changes. The least significant change (LSC) of TRACP 5b was calculated from data obtained from 15 patients with early BC.

**Results:**

Estrogen receptor status (Hazard Ratio (HR) = 0.397; *p *= 0.003) and visceral metastasis (HR = 0.492; *p *= 0.0045) were significantly correlated with OS. The OS was significantly shorter in those patients with higher baseline TRACP 5b activity based on a cut-off value to delineate the highest tertile (HR = 3.524; *p *< 0.0001). Further analysis demonstrated that among patients in the highest tertile, OS was significantly longer in those patients who had achieved a decrease of serum TRACP 5b activity greater than the LSC (38.59%) (*p *= 0.0015).

**Conclusions:**

We found that TRACP 5b activity and its interval change after treatment bore a prognostic role in BC patients with bone metastasis and a high baseline serum TRACP 5b activity. Further prospective phase II study is necessary to confirm these results.

## Background

Breast cancer (BC) is the most common malignancy in women. Once metastasis occurs, most patients become incurable. However, metastatic BC is a heterogeneous disease in which some women survive only a few weeks whereas others survive many years. Hormonal receptor status including estrogen receptor (ER) and progesterone receptor (PR) as well as human epidermal growth factor receptor 2 (HER2) have been shown to predict treatment response and survival [1]. Patients with metastasis only to bone survive longer than those with visceral metastasis [2]. Nevertheless, patients with bone metastasis still carry heterogeneous outcomes, and the survival time might become shorter when skeletal-related events (SRE) such as pathological fractures, radiation to bones, spinal cord compression, hypercalcemia, and bone surgery occur to patients [3]. Biomarkers of bone metabolism are eagerly sought as a way to predict outcome in BC patients with bone metastasis [3-5]. In a recent post hoc analysis from a randomized phase III trial comparing zoledronic acid and pamidronate in patients with bone metastasis, Lipton et al. demonstrated that early normalization of elevated baseline urinary N-telopeptide of type 1 collagen (NTX) levels was associated with longer event-free and overall survival (OS) times in BC patients receiving zoledronic acid[6,7]. Our previous studies demonstrated that NTX and tartrate-resistant acid phosphatase 5b (TRACP 5b) have similar clinical performance for the diagnosis of bone metastases and treatment monitoring [8]. Furthermore, preanalytical variables such as diurnal rhythm and feeding influence NTX and other collagen biomarkers more than TRACP 5b activity [8]. In most clinical practices it is difficult to standardize the time of blood draw. Therefore TRACP 5b has certain advantages over collagen biomarkers for assessment of bone metabolism.

TRACP 5b is an enzyme secreted by osteoclasts and its activity can be measured specifically in serum by immunoassay[9]. Serum TRACP 5b activity is a marker of osteoclast number and becomes elevated in the serum of individuals with increased bone turnover rate such as pre-pubertal children and post-menopausal women [9-11]. We and other investigators have found that serum TRACP 5b activity could become elevated in BC patients with bone metastasis because 80% of the bone metastasis lesions caused by BC are osteolytic in nature [12,13]. Furthermore elevated serum TRACP 5b activity in BC patients is positively correlated with the extent of bone metastasis [8,14]. We have also demonstrated that serum TRACP 5b activity can be potentially used as a marker to monitor treatment response in BC patients with bone metastasis and as an adjunct to bone scintigraphy for the diagnosis and follow-up of bone metastasis [8,14]. In the current study we hypothesize that an elevated serum TRACP 5b activity may reflect a higher tumor burden and further signify a worse outcome in BC patients with bone metastasis. Therefore we examine if baseline serum TRACP 5b activity and its interval change after treatment can be used as prognostic markers for overall survival time in BC patients with bone metastasis. Confounding factors including age, tumor markers including carcinoembryonic antigen (CEA) and CA 15.3, ER, HER2 over-expression, and the presence of visceral metastasis were adjusted.

## Methods

### Study population

We analyzed retrospectively biochemical and clinical data from 100 BC patients with newly diagnosed bone metastasis who had participated in our previous studies of serum TRACP 5b activity in BC [12,13]. ER and HER2 were stained by immunohistochemistry on paraffin-embedded tumor tissues. Expression levels were determined semi-quantitatively by a BC pathologist (CPY). Bone metastasis was diagnosed according to clinical symptoms, radiological images, and ^99m^Tc-hydroxy-methylene-diphosphanonate whole-body bone scintigraphy. All patients underwent chemotherapy, hormonal therapy, radiotherapy, and/or bisphosphonates therapy as clinically indicated. Fifteen informed, consenting subjects participated as a control group to determine the least significant change (LSC) of serum TRACP 5b activity. The control subjects were females aged 34 to 67 years with a median age of 50. All were in early stages of BC with no distant metastasis and undergoing adjuvant treatment as clinically indicated. All studies were performed under the guidelines of the Helsinki Declaration and approved by the Human Subjects Protection Offices (IRB) of Tri-Service General Hospital.

### Serum TRACP 5b Activity Assay

Serum from each patient had been collected and processed at diagnosis of bone metastasis and monthly thereafter for 6 months or until death as described in previous studies [12,13]. Osteoclastic TRACP 5b activity was measured by an antigen capture immunoassay as previously reported [8,14]. The clinical specificity and sensitivity of this assay for both osteoporosis and extensive bone metastasis in BC have been reported previously [14,15]. The analytic precision was estimated as the mean percent coefficient of variation (%CV) for duplicate measurements. The inter-assay error was determined by repetitive independent assay over a one-week period of aliquots of six sera ranging in activity from 2.54 to 9.37 μmol/min/L. The average CV was calculated to be 3.9%. The intra-assay error was determined by simultaneous assay of eight duplicates of five sera ranging in activity from 2.50 to 11.0 μmol/min/L; the average CV was calculated to be 5.1%.

### Statistical Analyses

All descriptive data are expressed as median (range). The dependent variable was OS in days or months. Cox proportional regression model (SAS 9.1.3) was used to assess the association of interest, as well to adjust the potential confounders of age, tumor marker (CEA, CA 15.3), ER and HER2 status, and visceral metastasis. To avoid co-linearity among the independent variables, co-linearity diagnostic analysis was done with the following criteria: tolerance >0.4 or variance inflation <2.5 and condition number <10. There was no co-linearity among independent variables. Patients were divided into 2 groups according to their scales of TRACP 5b activities, i.e. the top 1/3 and the bottom 2/3. Student *t *test and Chi-square test were used to assess the allocation between these two groups. The LSC is the minimum change of TRACP 5b activity considered to be of biological significance at a 95% level of confidence, a level of confidence normally used in clinical practice. In this study, we retrieved data from 15 randomly selected early breast cancer patients from previous studies to calculate the LSC of serum TRACP 5b from monthly samples taken over a 4-month period. The percent change in TRACP 5b was calculated according to the following formula: Δ = [(baseline data - subsequent data)/baseline data] × 100%. Cox proportional regression model was used to compare survival of patients in the highest tertile of baseline TRACP 5b activity to the remaining patients in the lower and middle tertiles. The maximal interval change within 6 months for serum TRACP 5b activity was used to stratify patients into two groups according to whether the interval change was greater or less than the LSC. Cox proportional regression model was used to estimate the significance and the hazard ratio of each variable in the total cohort, those in the highest TRACP 5b tertile, and those in the remaining group separately.

## Results

### Patient Characteristics

The demographics of the 100 BC patients with bone metastasis are shown in Table 1. They all were women ranging in age from 30 to 78 years with a median age of 50. The median OS was 481 days. Fifty-seven percent of patients had concurrent visceral metastases on the initial diagnosis of bone metastasis. Sixty-three of the 100 patients were ER-positive, 28 ER-negative, and 9 with unknown ER status. Eighteen of these patients were HER2-positive, 57 HER2-negative, and 25 had unknown HER2 status.

**Table 1:** Characteristics of the 100 breast cancer patients with bone metastasis

Female/male		100/0
Median age		50 (30-78)
Median time from initial diagnosis to enrollment		1007(2-6801)
Median overall survival time (Days)		481(1-4465)
Median initial TRACP-5b activity (U/L)		3.894 (1.21-24.245)
Median CEA (ng/ml)		3.5 (1-8165)
Median CA-153 (U/ml)		42.15 (6.59-3040)
ER	positive	63
	negative	28
	unknown	9
Her-2/neu	positive	18
	negative	57
	unknown	25
Visceral metastasis present		57
Bone metastasis only		43
Prior chemotherapy	None	18
	One line	46
	≥ 2 lines	23
	unknown	13

### Least Significant Change of TRACP 5b

The LSC of serum TRACP 5b activity over time was calculated from 4 consecutive monthly samples from 15 patients with early BC without distant metastasis. The LSC of TRACP 5b was 38.59%. This meant that a TRACP 5b change at months 1 to 4 of at least 38.59% was required to have a 95% level of confidence that a biologically significant change had occurred. If this change was a decline in serum TRACP 5b activity, it was considered to be a positive treatment response and clinical improvement.

### Serum TRACP 5b and Overall Survival Time

**Figure 1:**
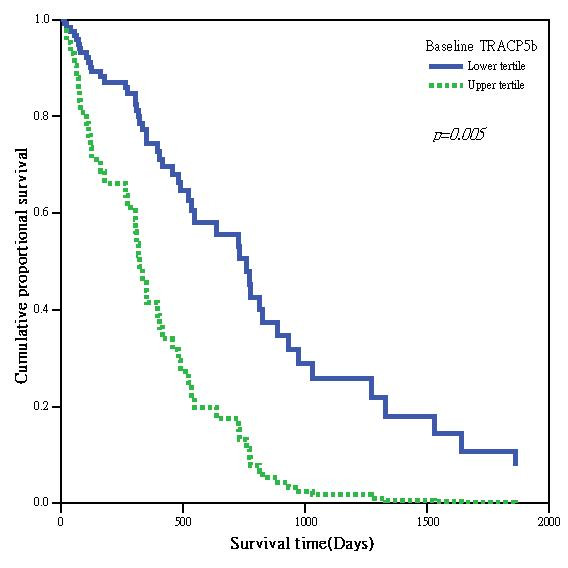
**Survival curve comparing patients with baseline TRACP 5b activity in the upper third and the lower two thirds using Cox-regression model**.

**Figure 2:**
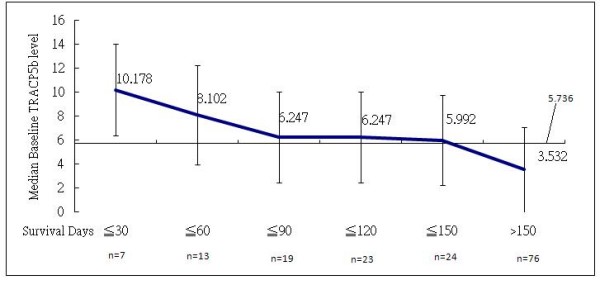
**The median baseline TRACP 5b activity (U/L) in patients with different overall survival time**.

Table 2 summarizes the results of Cox proportion regression models assessing the OS as a function of baseline variables including age, ER status, CEA, CA15.3, HER2 status and visceral metastasis in addition to baseline serum TRACP 5b activity. The presence of visceral metastasis in addition to bone metastasis as a dichotomous variable (Hazard ratio (HR) = 0.492; *p *= 0.0045) correlated inversely with the OS. ER positivity as a dichotomous variable correlated positively with a longer survival (HR = 0.397; *p *= 0.003). No other continuous biomarkers showed significant correlation with the OS. We divided the 100 patients into 2 groups arbitrarily according to the scale of serum TRACP 5b activity, i.e. those with the top 1/3 baseline activities (TRACP 5b ≥ 5.736 U/L) and the remainders. The demographics and allocation between these 2 groups are listed in Table 3. The OS in patients with the highest one-third of serum TRACP 5b activity was significantly longer (HR = 3.524; p < 0.001) than for those with the lower two-thirds of serum TRACP 5b (Table 2, Figure 1). We further calculated the median baseline serum TRACP 5b activities in different patient groups stratified by the OS. As shown in Figure 2, there was an inverse relationship between baseline serum TRACP 5b activity and OS.

**Table 2:** Cox-proportional regression analysis results of overall survival time of 100 breast cancer patients with bone metastasis.

					**Patients with available sequential TRACP 5b activities**							
					
	**Total**	**cohort**	**n = 100**		**Lower**	2/3*	**n = 54**		**Top**	1/3*	**n = 27**	
			
	HR**	CI**		p***	**HR**	**CI**		**p**	**HR**	**CI**		**p**
			
Age	ns****	-	-	ns	0.945	0.896	0.997	0.0384	ns	-	-	ns
												
Baseline TRACP 5b activity	3.524	2.066	6.010	< 0.001	-	-	-	-	-	-	-	-
												
ΔmaxTRACP 5b*****	-	-	-	-	ns	-	-	ns	0.213	0.082	0.555	0.0015
												
CEA	ns	-	-	ns	ns	-	-	ns	ns	-	-	ns
CA15.3	ns	-	-	ns	ns	-	-	ns	ns	-	-	ns
ER positivity	0.397	0.240	0.656	0.0003	0.260	0.103	0.655	0.0043	0.374	0.148	0.947	0.0379
HER-2/neu positivity	ns	-	-	ns	ns	-	-	ns	ns	-	-	ns
Visceral metastasis absent	0.492	0.301	0.802	0.0045	ns	-	-	ns	ns	-	-	ns

*. Two Group are classified by the baseline TRACP 5b activity in the top 1/3 (TRACP 5b ≥ 5.736 U/L) and bottom 2/3 (TRACP 5b < 5.736U/L)

**. HR = Hazard ratio; CI = Confidence interval

***. p = p value of each variant

****. ns= Not significant

*****. Maximal interval change of TRACP 5b as a dichotomous variable (Δmax TRACP 5b × 38.59% versus ≤ 38.59%).

**Table 3:** Demographic and allocation analysis between highest tertile and lower tertiles

		Top 1/3 (n = 33) (Median ± SD)	Bottom 2/3 (n = 67) (Median ± SD)	*p*-value
Age*		49.7 ± 8.48	50.0 ± 9.29	0.995
Survival time (days) *		202 ± 264	700 ± 702	<0.0001
Pre-enrolled time (days) *		817 ± 820	1218 ± 1584	0.021
Baseline TRACP 5b*		8.006 ± 3.921	3.063 ± 1.050	<0.0001
CEA*		5.34 ± 83.02	2.50 ± 1102	0.577
CA 15.3*		196.7 ± 648.06	32.59 ± 142.42	<0.0001
Visceral metastasis**	Positive	22	35	0.248
	Negative	11	32	
ER**	Positive	16	47	0.156
	Negative	12	16	
HER**	Positive	6	12	1.00
	Negative	19	38	
Prior chemotherapy **	None	4	14	0.619
	One line	16	30	
	≥ 2 lines	7	16	

*: Student *t *test (2-tailed)

**: Chi-Square

### Serum TRACP 5b Interval Changes and the Overall Survival Time

**Figure 3:**
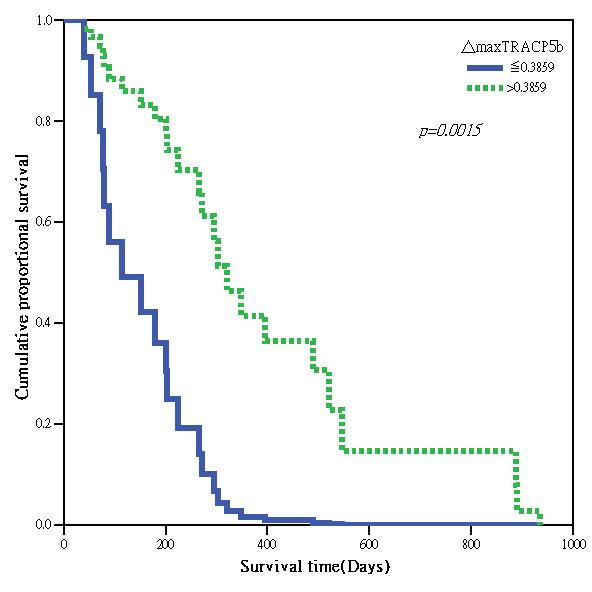
**Survival curve comparing patients with baseline TRACP 5b activity ≥ 5.736 U/L grouped by Δ_max_TRACP 5b > 38.59% and Δ_max_TRACP 5b ≤ 38.59% in a Cox-regression model**.

In the 100 patients studied, 81 had available serial serum TRACP 5b activity determinations. No subsequent TRACP 5b data could be retrieved from the remaining 19 patients, due to either early death or loss to follow up. The maximal interval change between baseline and follow-up serum TRACP 5b activities (Δ_max_TRACP 5b) within 6 months of enrollment in each individual patient was calculated as [(baseline TRACP 5b activity - TRACP 5b activity at maximal alteration)]/baseline TRACP 5b activity × 100%. The median time to the maximal change of TRACP 5b activity was 2 month. When the 81 patients' data were analyzed together, there was no correlation between Δ_max_TRACP 5b and the OS. However, when only patients with baseline serum TRACP 5b activities **in **the highest tertile (≥5.736 U/L) were analyzed (n = 27), Δ_max_TRACP 5b, stratified by LSC as a dichotomous variable, i.e. Δ_max_TRACP 5b > or ≤ 38.59%, significantly correlated with OS (HR = 0.213, *p *= 0.0015; Table 2 and Figure 3).

## Discussion

The survival of patients with metastatic BC varies from months to several years, and the most common metastatic site is bone. Metastasis to sites other than bone often dramatically shortens survival [2,4]. Bone metastasis is often related to ER-positivity and low grade tumor [16]. Our findings were consistent with previous studies in that ER positivity and bone-only were associated with a longer survival [17]. Tumor markers have been proven previously to have limited or no prognostic value [2,18], and they were not prognostic markers for survival in this study. Specifically HER2 status did not contribute as a prognostic marker perhaps due to the use of trastuzumab in half of our patients.

When marrow is invaded by BC cells, osteoclasts are recruited and differentiated at an increased rate by the production of high amounts of tumor-derived cytokines such as transforming growth factor β (TGF-β), interleukin-6 (IL-6), parathyroid hormone-related protein (PTHrP) [19]. It is reasonable to hypothesize that a higher tumor burden in the marrow could be associated with higher cytokine levels, which could further result in increased numbers of mature osteoclasts and elevated serum TRACP 5b activity.

We have already shown that serum TRACP 5b activity correlates with extent of bone metastasis in BC patients [13]. Since a higher tumor burden is related to a poorer survival, we tested the hypothesis that elevated serum TRACP 5b activity, as a measure of extent of bone disease, could be a marker of poorer prognosis in BC patients with bone metastasis. In our study, TRACP 5b activity was a significant prognostic marker for survival along with ER status and bone-only metastasis.

Nevertheless, serum TRACP 5b activity may not be elevated in all BC patients with bone metastasis, only in those with extensive metastasis [14]. Previous receiver operative characteristic curve analysis showed that, although serum TRACP 5b activity is highly specific for bone metastasis, its sensitivity is only 80% [12]. Other factors to weaken this association could be whether bone metastasis is osteoblastic, osteolytic or mixed. If the lesions were primarily osteoblastic, serum TRACP 5b activity might be lower than those with osteolytic or mixed lesions (Personal observations by TYC). Baseline bone metabolic rate of individual patients may also confound analysis because age and the presence of osteoporosis have been shown to have impact on serum TRACP 5b activity [12]. For example, serum TRACP 5b activity may be lower in pre-menopausal patients with bone metastasis. Although we corrected for age, BC and bone metastasis afflicts younger woman in Asia than in the West. Also in this regard, many post-menopausal women take anti-resorptive drugs to preserve bone health, which may cause apoptosis of osteoclasts and decrease the serum TRACP 5b activity [10]. All these confounding factors may have weakened the power of serum TRACP 5b activity in reflecting the tumor burden. In fact, some patients with a lower serum TRACP 5b activity may actually carry a high tumor burden.

Accordingly, we proposed that only in those patients with higher serum activity can TRACP 5b genuinely reflected higher tumor burden. Whereas in those patients with lower activities, serum TRACP 5b may not be useful as a prognostic factor. However, the real cutoff value to differentiate high from low serum TRACP 5b activity is unknown. Therefore we divided our patients into subgroups arbitrarily by the scale of baseline TRACP 5b activities, i.e. top 1/3 versus remaining 2/3 with the cutoff value of 5.736 U/L. We indeed found that those patients with baseline serum TRACP 5b activity higher than 5.736 U/L had a significantly shorter survival than the remainders.

Interval changes of bone markers after treatment in BC patients with bone metastases have been proposed as potential prognostic factors. Lipton *et al*. reported that a persistently elevated urinary NTX level was related to poorer prognosis [6,7]. Early normalization of baseline elevated NTX was associated with longer event-free and overall survival times. Additionally, an extremely elevated baseline urinary NTX was associated with increased tumor burden in bone and an extremely aggressive disease. Our results showed that serum TRACP 5b activity interval change might also be a potential prognostic marker, but it needs to be validated independently. In those patients with baseline serum TRACP 5b activities higher than 5.736 U/L, a Δ_max_TRACP 5b greater than LSC after treatment had significantly longer OS compared to the rest of the group after adjustment of other prognostic factors. Serum TRACP 5b activity of more than 5.736 U/L is probably related to a significant volume of cancer cells. Furthermore, when interval change of a biological marker is considered to be of prognostic value for a specific clinical setting, the LSC of a marker should be taken into account while interpreting the results. In our study, the LSC for serum TRACP 5b activity was 38.59% determined from 15 early BC patients. When we dichotomized those patients whose serum TRACP 5b activities were higher than 5.736 U/L into 2 groups based on response, we were able to demonstrate that patients with a Δ_max_TRACP 5b > 38.59% had a significantly longer survival than those without. However, this correlation was not existent when all patients were considered together. Other confounding factors such as ER status and bone-only metastasis correlated more strongly with the OS. Δ_max_TRACP 5b lost its statistical significance as a prognostic marker for survival in the lower tertile groups. Our explanation is that serum TRACP 5b may not reflect the real tumor burden in those patients with lower activities. This may have biased the result when all patients were included. Another possible explanation might be that Δ_max_TRACP 5b only correlated with treatment response, which was not a strong prognostic marker for OS.

There are several limitations of this study. Firstly this is a retrospective study and patients did not receive uniform treatment. Nevertheless, it does reflect the real context of clinical practice, and effects due to specific treatments were not our primary objective. Secondly, the patient numbers are relatively small and the results could not be interpreted by stratification. Thirdly, tissues were not adequate enough or not available for determining ER status in 9 patients and HER2 in 25 patients. Fourthly, the cut-off value of 5.736 U/L was arbitrarily determined by dividing patients into top 1/3 and bottom 2/3 according to the scale of serum TRACP 5b activity. This cut-off may not be the same in different laboratories and with different assays. Fifthly, 19 of the 100 patients in this study did not have serial TRACP 5b data to calculate the association between its interval change and the overall survival time. This may have biased the final results.

## Conclusion

Our study, deemed as a concept proving study, has shed light on the relative diagnostic and prognostic value of serum TRACP 5b in BC patients with bone metastasis. The research effort is exploratory and hypothesis-generating only, given the small sample size and certain study design limitations. To determine the true value of TRACP 5b as a prognostic marker for all patients with bone metastasis will require a prospective phase II study over a longer period including more patients to allow for more refined stratification.

## Competing interests

The authors declare that they have no competing interests.

## Authors' contributions

YYW analyzed the clinical information along with laboratory data and drafted the manuscript. CHK performed the statistical analysis. CPY reviewed the pathological slides. TYC designed and oversaw the progress of the study as well as finalized the manuscript. JCY and LTY gave criticisms to this manuscript. AJ set up the TRACP 5b immunoassay, critically reviewed and assisted in writing this manuscript. SHL and HYL carried out the biomarker immunoassays and were responsible for the quality assurance. All authors read and approved the final manuscript.

## Pre-publication history

The pre-publication history for this paper can be accessed here:

http://www.biomedcentral.com/1471-2407/10/158/prepub
